# Rectal cancer in patients with hereditary nonpolyposis colorectal cancer: Surgical management and survival outcomes

**DOI:** 10.1186/1897-4287-9-S1-P41

**Published:** 2011-03-10

**Authors:** Y Nancy You, Devki S Saraiya, Thuy M Vu, Jula Veerapong, Patrick M Lynch, Miguel A Rodriguez-Bigas

**Affiliations:** 1 Surgical Oncology, UT M.D. Anderson Cancer Center, Houston, TX 77030, USA; 2 Gastroenterology, Hepatology & Nutrition, UT M.D. Anderson Cancer Center, Houston, TX 77030, USA

## Background

Hereditary nonpolyposis colorectal cancer (HNPCC) is hallmarked by microsatellite instability. The prognosis of HNPCC-related colon cancer is well characterized, but little is known about rectal cancers. The aim of this study was to report the long-term outcomes of HNPCC-related rectal cancer where current-era multimodality therapy was utilized.

## Methods

Patients referred to our institution for either primary or recurrent rectal cancer between 1992-2010 were identified based on following inclusion criteria: 1) pathogenic germline mutation in DNA mismatch repair genes (MMR; n=19); 2) germline variants of uncertain significance but tumor studies suggestive of MMR (n=6); 3) suggestive tumor studies but negative germline testing (n=5); and 4) suggestive tumor studies but no germline testing (n=4). Patients were reviewed for clinical characteristics and treatments, and followed to death or last contact.

## Results

Among the 34 patients, 21 (62%) were female. The median age at diagnosis of rectal cancer was 40 (range: 20-72). In 28 patients (82%), this was the index cancer leading to the diagnosis of HNPCC, and in 22 patients (65%), this was their first malignancy. Only a minority satisfied Amsterdam I (21%) or Amsterdam II (21%) criteria, while nearly all (94%) met the revised Bethesda criteria. Pathogenic mutations included MLH1 (15%), MSH2 (32%) and MSH6 (9%). The majority (67%) presented with locally advanced (T3/T4 and/or node positive, n=20) or metastatic disease (n=3), and 50% received neoadjuvant radiation with 5-FU based chemotherapy. Final pathologic stages are as outlined below. Patients underwent proctectomy (65%), total/near total coloproctectomy (21%), transanal excision (9%), and chemoradiation only (3%). Multivisceral resection was required in 9 patients (28%) and adjuvant therapy was given in 24 (71%). After a median followup of 4.1 years, 94% were alive. Six patients developed local-regional (n=3) or distant (n=3) disease recurrence, and 5 underwent successful surgical salvage. Metachronous CRC was found in 4 patients (12%) after a median of 8 years (range: 3.2-17), and all were amenable to surgical resection. The estimated 5-year freedom from recurrent or metachronous CRC was 76%. The 5-year overall survival was 93%, which was preserved at 10-years. Table 1.

**Table 1:** 

Pathologic Stage	After neoadjuvant (n=17)	No neoadjuvant (n=17)	Total (n=50)
0	5 (29%)	2(12%)	7 (21%)
I	2 (12%)	6 (35%)	8 (24%)
II	4 (24%)	5 (29%)	9 (26%)
III	5 (29%)	2 (12%)	7 (21%)
IV	1 (6%)		1 (3%)
Unknown		2 (12%)	2 (5%)

## Discussion

Rectal cancer may present as the index cancer for HNPCC over a wide age range. Despite advanced stages at presentation, excellent long-term prognosis can be expected with aggressive multimodality therapy. Vigilant surveillance for recurrent or metachronous CRC should be carried out over a prolonged time period to allow for repeat surgical salvage and preserved long-term survival.

